# miR-369-3p modulates inducible nitric oxide synthase and is involved in regulation of chronic inflammatory response

**DOI:** 10.1038/s41598-020-72991-8

**Published:** 2020-09-29

**Authors:** Viviana Scalavino, Marina Liso, Elisabetta Cavalcanti, Isabella Gigante, Antonio Lippolis, Mauro Mastronardi, Marcello Chieppa, Grazia Serino

**Affiliations:** National Institute of Gastroenterology “S. de Bellis”, Research Hospital, Via Turi, 27, Castellana Grotte, 70013 Bari, Italy

**Keywords:** miRNA in immune cells, Dendritic cells

## Abstract

Dendritic cells are the most important antigen-presenting cells that link the innate and acquired immune system. In our previous study, we identified that the upregulation of miR-369-3p suppresses the LPS-induced inflammatory response, reducing C/EBP-β, TNFα and IL-6 production. With the aim of gaining further insight into the biological function of miR-369-3p during acute inflammatory response, in the present study we identified novel gene targets of miR-369-3p and demonstrated the suppressive ability of these genes on the inflammatory dendritic cells. Bioinformatic analyses revealed that iNOS is a potential target of miR-369-3p. We demonstrated that the ectopic induction of miR-369-3p markedly reduced iNOS mRNA and protein as well as NO production. Moreover, we found that the upregulation of miR-369-3p decreased the release of TNFα, IL-6, IL-12, IL-1α, IL-1β in response to LPS, and increased the production of anti-inflammatory cytokines such as IL-10 and IL-1RA. In addition, LPS-induced nuclear translocation of NF-kB was inhibited by miR-369-3p. Levels of miR-369-3p were decreased in human inflamed regions of human intestine obtained from IBD patients. Our results provide novel additional information on miR-369-3p as a potential core of the signaling regulating the inflammatory response. These findings suggest that miR-369-3p should be considered as a potential target for the future development of new molecular therapeutic approaches.

## Introduction

Dendritic cells (DCs) are the most potent antigen-presenting cells (APCs) able to initiate the innate and adaptive immune system^1^. The recognition of pathogen-associated molecules by antigen-presenting cells is crucial step to the initiation of the immune response^2^ resulting in DC maturation, characterized by the expression of numerous cell surface molecules. These include increased major histocompatibility complex class II (MHC II) and co-stimulatory molecules (CD80, CD86 and CD40) and the production of soluble factors like cytokines and chemokines^3^. Following DCs activation, high levels of nitric oxide (NO) are produced due to an upregulation of inducible nitric oxide synthase (iNOS) and an activation of Nuclear Factor kappa-light-chain-enhancer of activated B cells (NF-κB) pathways^4^.

Mucosal resident DCs are frequently in close contact with foreign antigens, but are educated by the host tissue to become inflammatory resistant^5,6^. Uncontrolled DCs-mediated inflammation is involved in the cascade of events leading to chronic inflammatory diseases, such as inflammatory bowel disease (IBD)^7^. Recent studies have investigating the possibility of using immunomodulatory factors to inhibit DC inflammatory maturation to be adopted as complementary treatment for conventional therapy for IBD. Among these, nutrition-derived polyphenols have been widely studied^8,9^, but due to chemical limitation, their administration rarely achieved clinically relevant results^10^. For this reason, we recently investigated and identified a signature of miRNAs specific of DCs exposed to quercetin^11^.

miRNAs are a class of small endogenous non-coding RNA that post-transcriptionally regulate gene expression. miRNAs have emerged as key regulators of many cellular processes, including immunity^12–14^. In our study, we demonstrated that miR-369-3p, regulating C/EBP-β, TNFα and IL-6, is responsible for the anti-inflammatory effect of quercetin in bone marrow-derived dendritic cells (BMDCs). Moreover, similarly to the results of quercetin exposure, we confirm the protective role of miR-369-3p in DCs stimulated by LPS in suppressing the LPS-induced inflammatory response^11^.

With the aim to gaining further insight into the biological function of miR-369-3p during acute inflammatory response, in the present study, we identified novel gene targets of miR-369-3p. We demonstrate the suppressive ability of miR-369-3p to reduce the LPS-mediated dendritic cells inflammatory response. In particular, we focused our attention on the generation of NO, iNOS, cytokines and also on investigating the activation of NF‑κB signaling. These findings unravel an alternative regulatory pathway for miR-369-3p, paving the way for a therapeutic translation of miR-369-3p for chronic inflammatory syndromes.

## Results

### In silico analysis of targets

**Figure 1:**
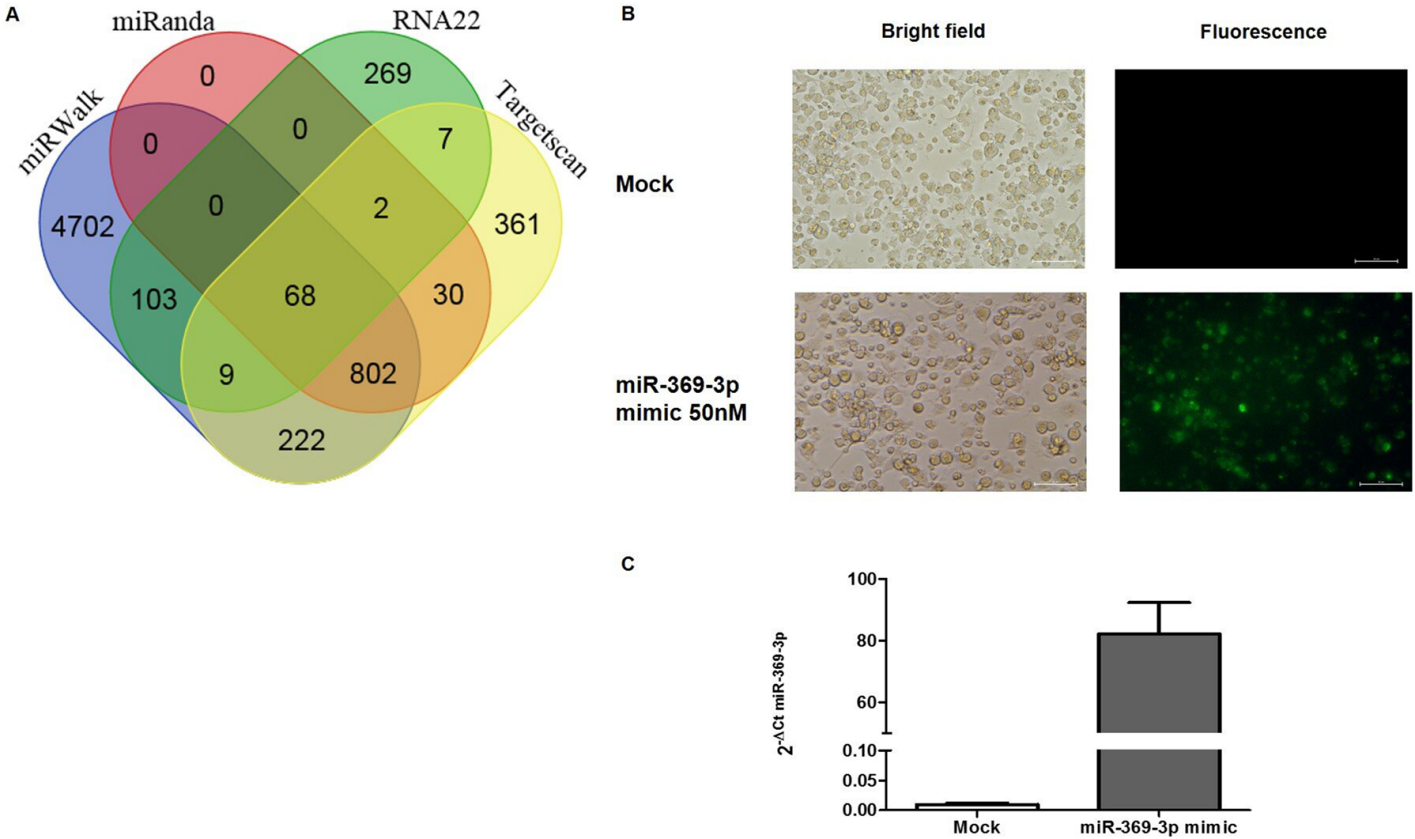
Transfection efficiency of BMDCs. BMDCs were transfected with 50 nM of FAM-labeled miR-369-3p mimic and unlabeled miR-369-3p mimic. (**A**) Representative images of BMDCs transfected with FAM-labeled miR-369-3p mimic. Bright‑field (left) and fluorescence (right) images for mock condition and transfected condition were acquired using fluorescence microscopy. Original magnification, × 20. Scale bar presents 50 μm. (**B**) miR-369-3p expression levels in transfected BMDCs. miR-369-3p levels were determined by qRT-PCR and normalized to the geometric mean of two endogenous controls (miR-186-5p and miR-26a-5p). Data are shown as the means ± SEM.

To find other molecular target of miR-369-3p, we performed a bioinformatic analysis focusing our attention not only on the 3′ UTR but also the 5′ UTR and CDS regulatory regions. We found that miR-369-p had one putative target located in CDS region of NOS2 gene. This putative target was predicted by four different algorithms as miRWalk, miRanda, RNA22 and TargetScan (Fig. 1A).

### miR-369-3p decreases iNOS expression

In order to validate the bioinformatic analysis, we performed transient transfection experiments with 50 nM of the miR-369-3p mimic using LPS-stimulated BMDCs from 4 wild-type mice. Firstly, we evaluated the efficiency of transfection using a fluorescently labeled miR-369-3p mimic (Fig. 1B). Then, we quantified the expression levels of miR-369-3p in mock condition and miR-369-3p-transfected DCs (Fig. 1C). We found that the transfection of miR-369-3p in DCs increased miR-369-3p expression.

**Figure 2:**
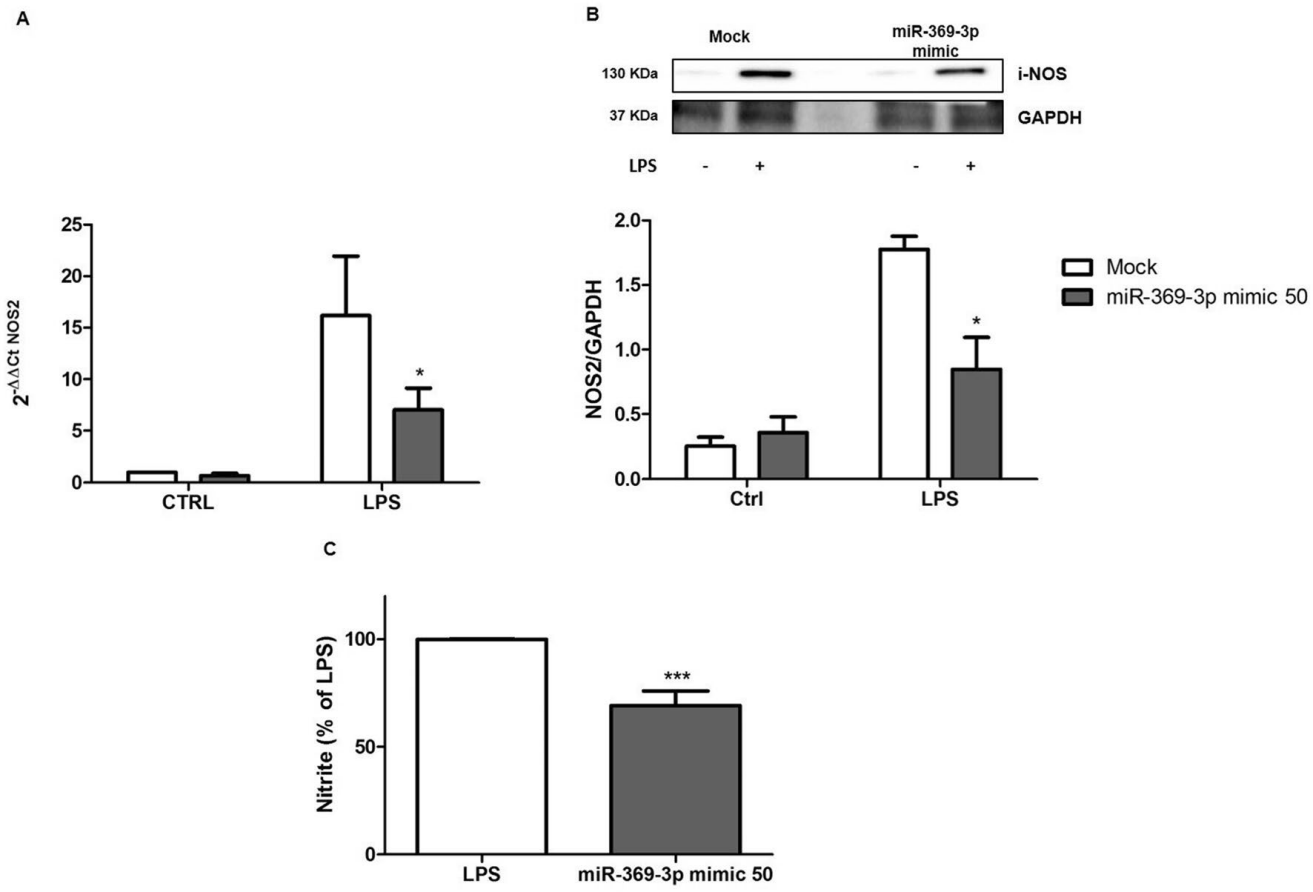
miR-369-3p regulates iNOS mRNA and protein expression and LPS-induced NO production. (**A**) The mRNA expression levels of iNOS were evaluated by qRT-PCR in LPS-stimulated BMDCs transfected with 50 nM of miR-369-3p mimic. The ectopic increase of endogenous miR-369-3p in LPS-stimulated BMDCs led to a significant decrease of iNOS (2.3-fold decrease). (**B**) Expression analysis of iNOS protein after miR-369-3p mimic transfection using Western blot analysis. A significant decrease in iNOS protein production was shown after transfection. “Mock” indicates mock-transfected cells going through the transfection processes without the addition of mimic miRNA. The housekeeping gene was used to normalize expression data. Ratio was calculated by dividing the normalized values of stimulated and/or transfected cells by the values of normalized control cells. Data are representative of four independent experiments. The histograms represent the mean ± SEM. *p < 0.05 compared to the same treatment in the mock condition. The presented blots were cropped. Full-length blots are shown in Supplementary Fig. S1. (**C**) miR-369-3p induction reduces NO production. LPS-stimulated BMDCs after miR-369-3p mimic transfection showed a significant reduction of NO production. Nitrite was measured in the culture supernatants using Griess reagent and expressed as a percentage of the LPS group. *p < 0.05 compared to LPS group.

Afterwards, we investigated the effect of miR-369-3p on NOS2 expression. The increase of endogenous miR-369-3p led to a significant decrease of iNOS mRNA expression in LPS-treated BMDCs compared to mock control (p < 0.05; Fig. 2A).

In addition, we also tested whether miR-369-3p was able to control iNOS protein expression. BMDCs were transiently transfected with 50 nM of miR-369-3p mimic and then stimulated with LPS. Western blot analysis showed that the transfection with miR-369-3p mimic resulted in a significant decrease of iNOS protein production (Fig. 2B).

### Effect of miR-369-3p on LPS-induced NO production

To assess the effect of miR-369-3p induction on influencing NO production, we measured nitrite release in LPS- stimulated BMDCs after transient transfection with 50 nM of miR-369-3p mimic molecules. We found that the increased intracellular amount of miR-369-3p caused a significant reduction of NO release in LPS-stimulated BMDCs compared to mock control (p < 0.001; Fig. 2C).

### Effects of miR-369-3p on LPS‑induced inflammatory cytokine release

**Figure 3:**
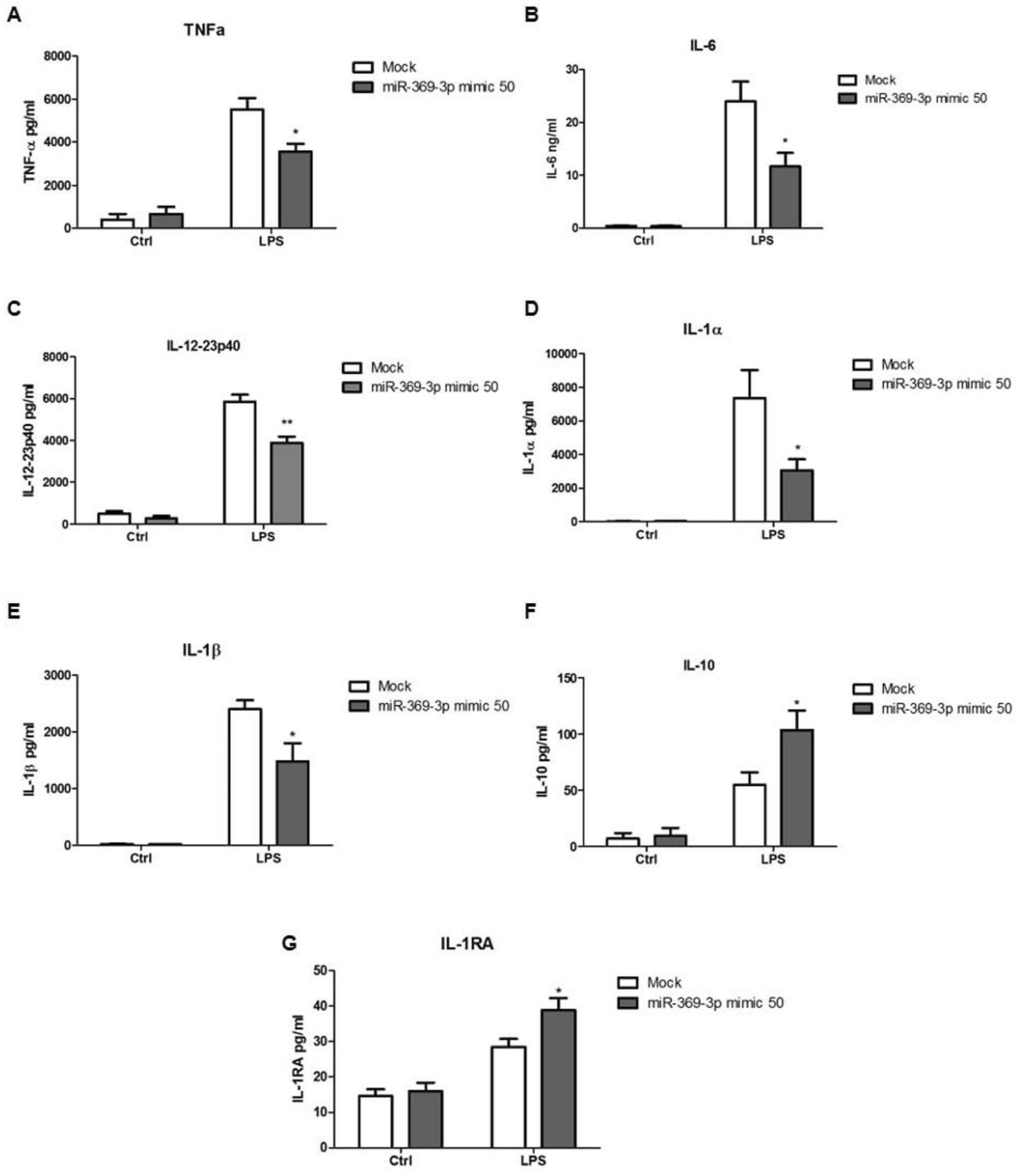
miR-369-3p is a negative regulator in LPS-induced inflammatory response in BMDCs. miR-369-3p upregulation in LPS-stimulated BMDCs after mimic transfection led to a significant decrease of TNFα (**A**), IL-6 (**B**), IL-12 (**C**), IL-1α (**D**), IL-1β (**E**) and increase of IL-10 (**F**) and IL-1RA (**G**) production in response to LPS. The secretion of cytokines was analyzed by ELISA 24 h after LPS stimulation. “Mock” indicates cells going through the transfection processes without the addition of mimic miRNA. Data are representative of four independent experiments. The histograms represent the mean ± SEM. *p < 0.05 compared to the same treatment in the mock condition.

To investigate whether the expression of miR-369-3p was functionally involved in the production of cytokines induced by LPS, we induced miR-369-3p expression by transiently transfection of DCs with miR-369-3p mimic molecules. Transfected DCs were then stimulated with LPS for 24 h and the levels of different cytokines production were analyzed in the cell-free supernatants. miR-369-3p induction significantly decreased TNFα, IL6, IL-12, IL-1α, IL-1β production in response to LPS (p < 0.05, Fig. 3A–E). On the contrary, the production of other anti-inflammatory cytokines such as IL-10 and IL-1RA was increased in miR-369-3p mimic transfected DCs after LPS stimulation compared with mock condition (p < 0.05, Fig. 3F,G).

These results confirm the role of miR-369-3p as an anti-inflammatory agent.

### Effects of miR-369-3p on the activation and nuclear translocation of NF‑κB in LPS‑stimulated dendritic cells

**Figure 4:**
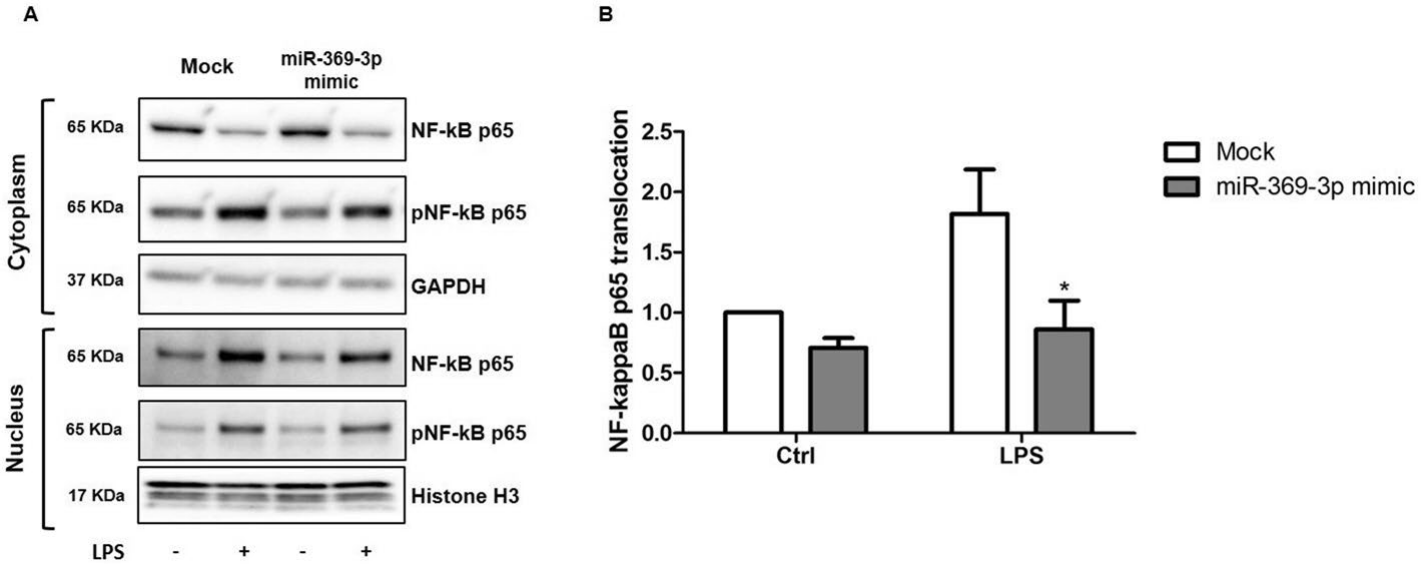
miR-369-3p had effect on the activation and nuclear translocation of NF‑κB in LPS-stimulated dendritic cells. (**A**) Cytosolic and nuclear extracts from LPS-stimulated DCs after transient transfection of miR-369-3p mimic were prepared. Western-blot analysis for total and phosphorylated NF‐κB p65 were assessed in both cytosolic and nuclear protein compartments. (**B**) A significant decrease in NF‐κB p65 translocation from the cytosol to the nucleus was observed after miR-369-3p transfection. The histograms represent the mean ± SEM of the NF‐κB p65 translocation index, expressed as a ratio of nuclear NF‐κB p65 content over cytoplasmatic NF‐κB p65 content. Data are representative of three independent experiments. The histograms represent the mean ± SEM. *p < 0.05 compared to the same treatment in the mock condition. The presented blots were cropped. Full-length blots are shown in Supplementary Fig. S2.

NF-κB is a major transcription factor that is activated during the inflammatory response to LPS regulating the expression of target genes involved in the activation of immune response in DCs^15^. NF-κB allocates in cytoplasm, while activated NF-κB subunits form dimers and translocate into nucleus to initiate the transcription of target genes. In order to investigate the involvement of miR-369-3p in the activation and translocation of NF-κB p65, we prepared cytosolic and nuclear extracts from LPS-stimulated DCs after transient transfection of miR-369-3p mimic. As shown in Fig. 4A, LPS‐induced NF‐κB activation, verified on the basis of increased phosphorylation level of cytosolic NF‐κB p65, was significantly reversed by the overexpression of miR‐369-3p. Moreover, we observed an inhibition of the translocation of NF‐κB p65 from the cytosol to the nucleus (Fig. 4B).

### LPS-mediated co-stimulatory molecules upregulation is not inhibited by miR-369-3p

**Figure 5:**
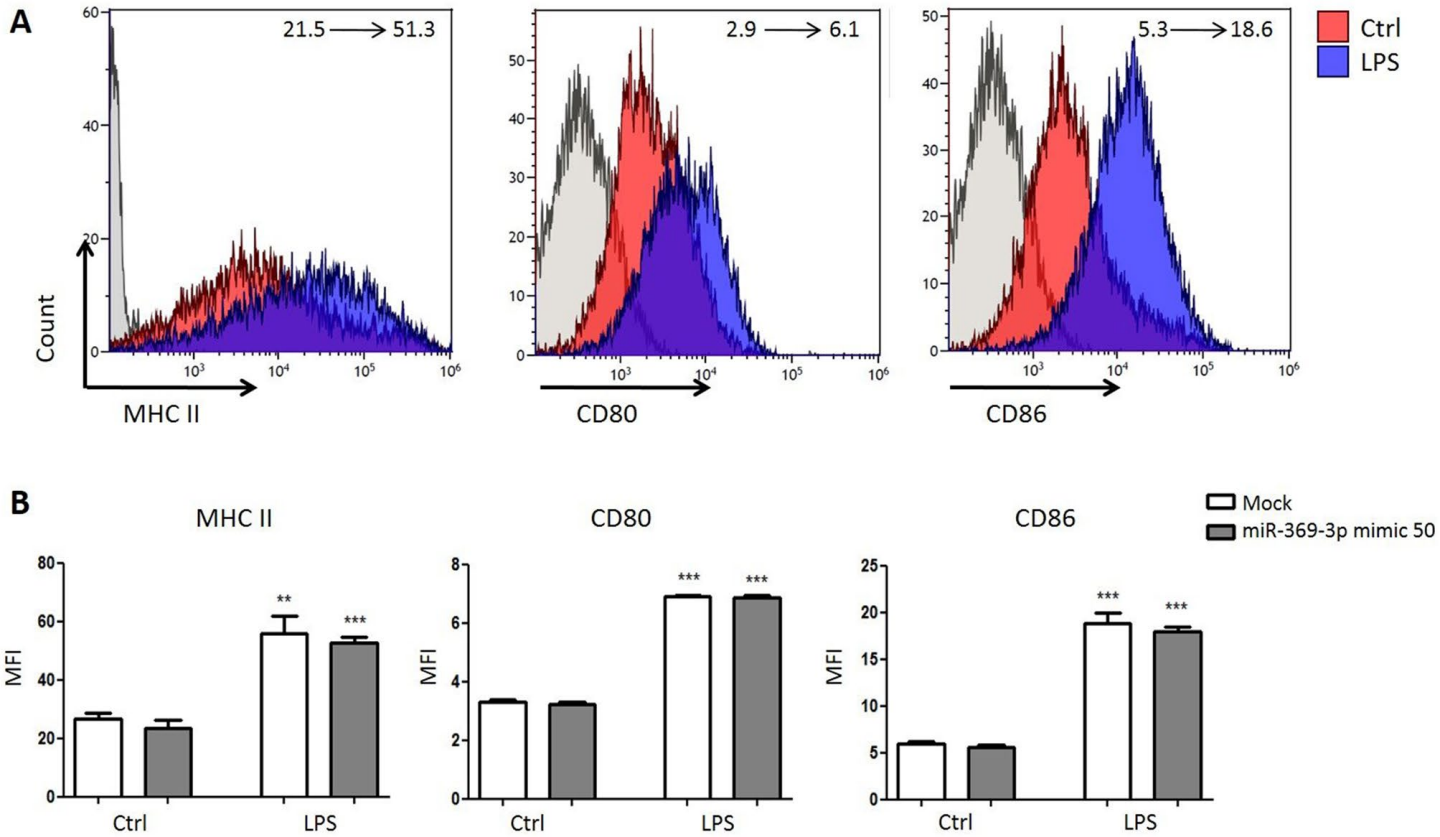
Effect of miR-369-3p on the expression levels of MHC class II, CD80, and CD86 were determined by flow cytometry. All data shown were gated on CD11c+ cells. The change of mean fluorescence intensity between the condition without LPS and condition with LPS is indicated. **p < 0.01; ***p < 0.001 (1-way ANOVA).

Using flow cytometry, we analyzed the expression levels of MHC class II and costimulatory molecules CD80 and CD86 in LPS-stimulated DCs after transient transfection of miR-369-3p mimic. Raising the amount of miR-369-3p intracellular levels did not affect the percentage and quantity of MHC class II, CD80 and CD86 in untreated or LPS exposed DCs (Fig. 5). This is not surprising, as the cell surface expression of co-stimulatory molecules is induced also in inflammatory impaired DCs^16^.

### miR-369-3p expression in human inflamed intestinal tract of IBD patients

**Figure 6:**
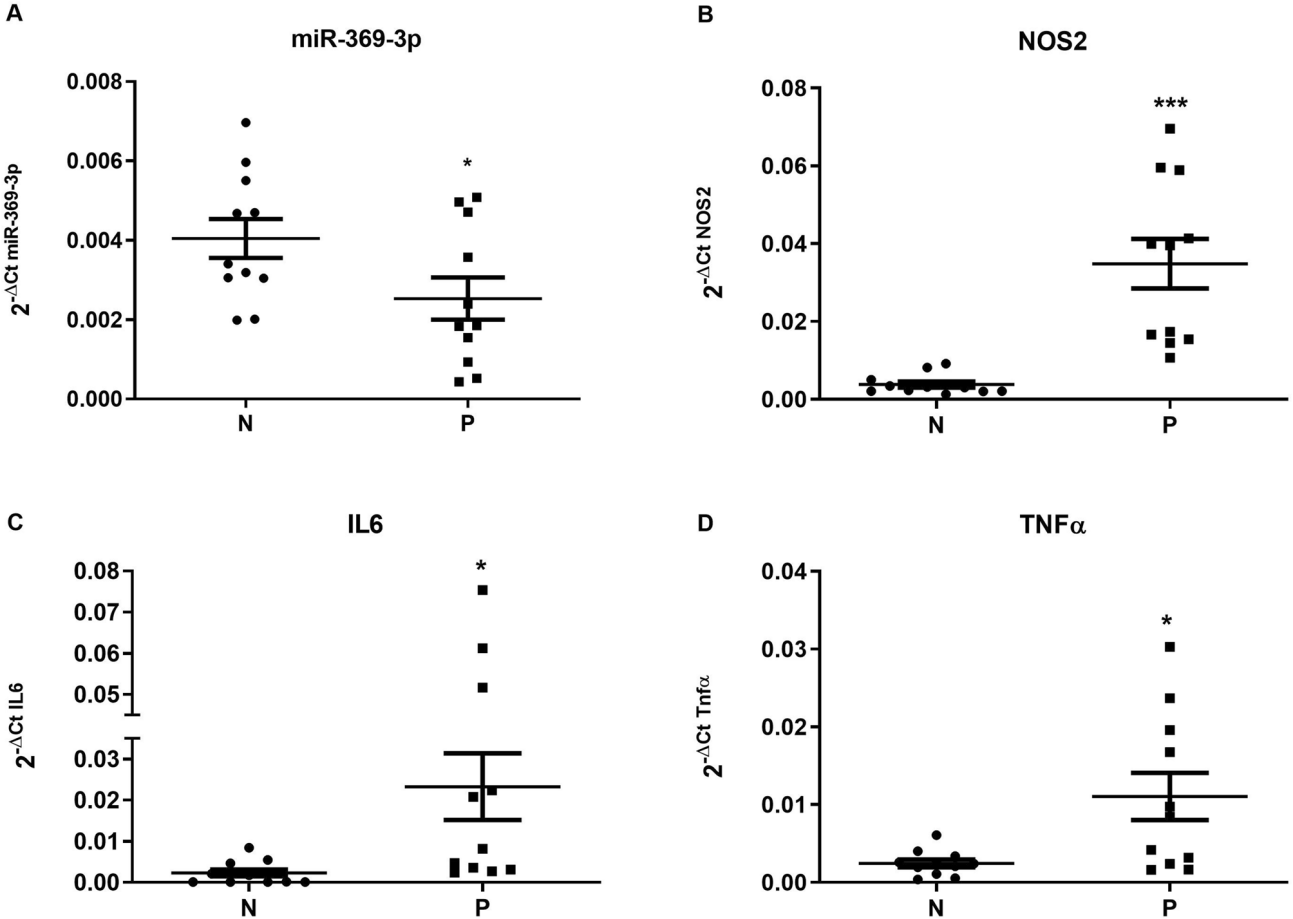
miR-369-3p, NOS2, IL6 and TNFα expression levels in non-inflamed and inflamed regions of intestine from IBD patients. Total RNA samples isolated from non-inflamed (N) and inflamed (P) regions of intestine were obtained from 11 IBD patients and were analyzed for miR-369-3p (**A**), NOS2 (**B**), IL6 (**C**) and TNFα (**D**) by qRT-PCR. miR-369-3p in the inflamed areas was significantly lower than those in non-inflamed tissue. Expression data of miR-369-3p were normalized to the endogenous control miR-26a-5p. NOS2, IL6 and TNFα expression levels were normalized to the housekeeping gene Gapdh. The dot-plot graphs represent the mean ± SEM. *p < 0.05; ***p < 0.0001.

In our previous paper^11^, we demonstrated that miR-369-3p play a role in regulating intestinal inflammation in murine colon tissue and intestinal dendritic cells. To support a role of miR-369-3p in the regulation of the inflammatory cascade in human, we determined the expression levels of miR-369-3p in tissue from 11 IBD patients analyzing RNA samples from non-inflamed and inflamed regions of intestine obtained from these patients. miR-369-3p levels in the inflamed areas were significantly lower than those in non-inflamed tissue (Fig. 6A). On the contrary, NOS2, IL6 and TNFα resulted significantly increased in inflamed areas compared to non-inflamed tissue (p < 0.05, Fig. 6B–D). This result confirms the involvement of miR-369-3p in regulating the inflammatory cascade, specifically in inflammatory bowel disease.

## Discussion

In the last years, miRNAs have emerged as key regulators of the inflammatory response in innate immune cells^12,17^. Several previous studies have underlined the importance of miRNAs in the regulation of DCs development and function^17^. The most widely investigated miRNA involved in DC activation is miR-155. This miRNA was highly upregulated after LPS stimulation. miR-155, by targeting SOCS1 and SHIP1, was a positive regulator of the production of several pro-inflammatory cytokines including IL-6, IL-23, IL-12, and TNFα^18^. Another miRNA induced by LPS stimulation was miR-146a that acts as negative regulator of DCs activation. miR-146a, by modulating two key molecules of the TLR4/NF-κB pathway, TRAF6 and IRAK-1, promote inflammation stimulated by LPS^19^. Zhu et al. reported that miR-181a-5p regulated the maturation of DCs induced by high mobility group box-1 protein^20^.

In our recent study, we identified a specific signature of 113 miRNAs that were differentially regulated in LPS-stimulated BMDCs after quercetin exposure, along with their candidate target genes. Among these miRNAs, we mechanistically demonstrated that the induction of miR-369-3p is able to downregulate the inflammatory response induced by LPS, mimicking the effect of quercetin exposure^11^.

In the current study, we gained further insight into the biological function of miR-369-3p during an acute inflammatory response, demonstrating that miR-369-3p regulates the levels of iNOS transcripts and protein in DCs.

We demonstrated that in DCs stimulated with LPS, the ectopic induction of miR-369-3p was associated with a reduced secretion of proinflammatory molecules (TNFα, IL-6, IL-12, IL-1α, IL-1β) and an increase of anti-inflammatory cytokines (IL-10, IL-1RA).

iNOS is responsible of a large amount of NO prolonged production induced by bacterial products and inflammatory cytokines in DCs and various cell types^21^. Here, we demonstrated that increased intracellular levels of miR-369-3p markedly reduce the LPS-induced production of NO by DCs.

iNOS is among the main downstream genes of NF-κB, but, in turn, iNOS is able to promote and inhibit NF-κB activity^22^. In fact, several studies have demonstrated that the inhibition of iNOS by gene interference or by some substances decreases activity^23–25^. NF-κB (p50/p65) in inactive form is located in cytosol, associated with the inhibitor protein, IκB-α. After stimulation with LPS or cytokines, IκB-α is phosphorylated and degraded, leading to the consequent activation of p50/p65 heterodimer, that translocate into the nucleus to activate a set of target genes related to inflammation^26^. In this context, in the present study, we have shown that miR-369-3p is able to reduce p65 translocation from the cytosol to the nucleus. In line with the reduction in NF-kB nuclear translocation, miR-369-3p treated DCs reduce the secretion of inflammatory mediators. This is important to suppress the adaptive arm of the inflammatory response^27^, even if the persistent expression of co-stimulatory molecules suggests that these topics warrant further investigation.

Our findings extend and deepen the knowledge on the role of miR-369-3p in modulating inflammatory processes. Overall, our data suggest that miR-369-3p plays a key role in negatively regulating the LPS-induced DCs responses mainly targeting iNOS. These findings identify miR-369-3p as a potential core player in the signaling that regulates the inflammatory response.

The involvement of miR-369-3p in the regulation of the inflammatory cascade in inflammatory bowel disease has also been demonstrated in human biopsies. Furthermore, a downregulation of miR-369-3p has been previously reported in Crohn’s disease plasma compared with control plasma^28^. Thus, we speculate that ectopic induction of this miRNA in intestinal tissue could revert the intestinal inflammation and so could beneficially be used for the treatment of IBD patients. Future studies will be needed to determine in mice models the efficacy of miR-369-3p administration in the treatment of chronic inflammatory diseases such as IBD. However, the results of our present and previous studies collectively suggest that miR-369-3p should be considered a potential target for the future development of new molecular therapeutic approaches.

## Materials and methods

### Mice

Our studies were conducted in accordance with national and international guidelines and were approved by the authors’ institutional review board (Organism For Animal Wellbeing [OPBA]). All animal experiments were carried out in accordance with Directive 86/609 EEC, enforced by Italian D.L. n. 116/1992, and approved by the Committee on the Ethics of Animal Experiments of the Ministero della Salute-Direzione Generale Sanità Animale (Prot.760/2017-PR released on 10/10/2017) and the official RBM veterinarian. Animals were sacrificed if found to be in severe clinical conditions during the experimental period to avoid suffering.

### Preparation and cultures of murine dendritic cells (DCs)

Bone marrow dendritic cells (BMDCs) were collected from C57BL/6 mice sacrificed at the age of 6- to 8-weeks. Tibias and femurs were isolated and temporarily stored in RPMI-1640 (Thermo Fisher Scientific, MA, USA). Bone marrows (BMs) were extracted by flushing with PBS with 0.5 mM EDTA (Bioscience Lonza, Basel, Switzerland) and red blood cells were lysed with Ammonium-Chloride-Potassium lysing buffer (ACK; Thermo Fisher Scientific, MA, USA). Using 10 ml dishes, 10 × 10^6^ cells were plated in RPMI-1640 (Thermo Fisher Scientific, MA, USA) supplemented with 10% heat-inactivated Fetal Bovine Serum (FBS; Thermo Fisher Scientific, MA, USA), 1% 1 M HEPES (Sigma-Aldrich, St. Louis, USA), 1% non-essential amine acids (Corning, USA), 1% 100 mM sodium pyruvate (Sigma-Aldrich, St. Louis, USA), 1% 10,000 µg/ml streptomycin and 10,000 U/ml penicillin (Thermo Fisher Scientific, MA, USA), 25 ng/ml IL-4 (Miltenyi Biotec, Bergisch Gladbach, Germany) and 25 ng/ml GM-CSF growth factors (Miltenyi Biotec, Bergisch Gladbach, Germany) and stored at 37 °C and 5% CO2. On day 5, BMDCs were harvested and plated in 12-well plates in RPMI-1640 complete medium without penicillin/streptomycin at the concentration of 1 × 10^6^/well BMDCs for cytofluorimetry analysis and 2 × 10^6^/well BMDCs for protein analysis. On day 10, BMDCs were transfected with miR-369-3p mimic at 50 nM concentrations (Qiagen, Hilden, Germany). On day 12, BMDCs were stimulated with 1 µg/ml of LPS (Sigma-Aldrich, St. Louis, USA) for 24 h.

### In vitro mimic transfection

On day 10, BMDCs were transfected with 50 nM of miR-369-3p mimic concentrations (Qiagen, Hilden, Germany) using TKO transfection reagent (Mirus Bio LLC, WI, USA), in accordance with the manufacturer’s instructions. Each transfection experiment was associated with mock control, where cells were transfected only with TKO transfection reagent (Mirus Bio LLC, WI, USA) without mimic, and with whole-cells control. The transfection efficiency was evaluated using a validated FAM-labeled miR-369-3p mimic (Thermo Fisher Scientific, MA, USA). In addition, the mirVana miRNA Mimic miR-1 Positive Control (Thermo Fisher Scientific, MA, USA) and the mirVana miRNA Mimic Negative Control #1 (Thermo Fisher Scientific, MA, USA) were used as positive and negative control, respectively. Bright‑field and fluorescence images were acquired using fluorescence Eclipse Ti2 microscope (Nikon Inc., Melville, NY). On day 12, BMDCs were stimulated whit 1 µg/ml LPS (Sigma-Aldrich, St. Louis, USA) and stored at 37 °C and 5% CO_2_ for 24 or 48 h.

### Total RNA extraction and quantitative real-time RT-PCR (qPCR)

Transfected BMDCs were used for total RNA extraction using TRIzol reagent (Invitrogen, Carlsbad, CA) according to the manufacturer's protocol. Total RNA was then resuspended in ribonuclease-free water. The RNA concentration was determined with the NanoDrop Spectrophotometer (Thermo Fisher Scientific, MA, USA).

cDNAs were synthesized with iScript Reverse Transcription Supermix (BioRad Laboratories, CA, USA) following the manufacturer’s recommendations.

qPCR amplification reactions were performed using SsoAdvanced Universal SYBR Green Supermix (BioRad Laboratories, CA, USA) and QuantiTect Primer Assay for Nos2 and Gapdh (Qiagen, Hilden, Germany). qPCR was conducted on a CFX96 System (Biorad Laboratories, CA, USA). The relative expression of Nos2 was normalized using the Gapdh gene amplification as reference standard. Comparative real-time PCR was performed in triplicate in 25 μl of final volumes, including no-template controls. Relative expression was calculated using the 2^−∆∆Ct^ method.

### Nitrite oxide quantification

Nitric oxide production was determined by measuring its end product, nitrite, using a Griess reagent (Thermo Fisher Scientific, MA, USA) according to manufacturer’s indications. Absorbance was measured at 548 nm by microplate reader and nitrite concentrations were estimated using a standard nitrite curve.

### Western blot

Total proteins were extracted with RIPA Buffer (Thermo Fisher Scientific, MA, USA) supplemented with cocktail proteinase inhibitors (Sigma-Aldrich, St. Louis, MO, USA) and quantified by the Bradford protein assay (Biorad Laboratories, CA, USA). For each sample, 50 µg of proteins were incubated with Sample Buffer Leammli 2x (Biorad Laboratories, CA, USA) and denatured at 100 °C for 5 min. Subsequently, samples were separated on Criterion TGX Stain-Free Gels, 4–20% (Biorad Laboratories, CA, USA) then transferred to Polyvinylidene Difluoride membranes (PVDF; Biorad Laboratories, CA, USA). The membranes were incubated with I-Bind Flex FD Solution (Thermo Fisher Scientific, MA, USA) at room temperature. The primary and secondary antibodies and washing solution were prepared and loaded with PVDFs on I-Bind Flex Western Device (Thermo Fisher Scientific, MA, USA) as reported in the manufacturer’s protocol. Immunocomplexes were detected with an enhanced chemiluminescence method (Biorad Laboratories, CA, USA) using a Chemidoc System (Biorad Laboratories, CA, USA) setting the acquisition protocol on automatic exposure. Signals were quantified by Image J program and the corresponding values were normalized with housekeeping proteins.

Cytoplasmic and nuclear extracts were obtained with NE-PER lysis buffers according to the supplier’s instructions (NE-PER Nuclear and Cytoplasmic Extraction Reagents (Thermo Fisher Scientific, MA, USA).

Primary antibodies and the conditions used for analysis include i-NOS (Cell Signaling #13120), NF-kB p65 (Cell Signaling #8242), pNF-kB p65 (Cell Signaling #3033), Histone H3 (Cell Signaling #4499), GAPDH (Santa Cruz sc-47724). Secondary antibodies used include Goat Anti-mouse IgG-(H+L)-HRP conjugate (Biorad Laboratories, CA, USA) and Goat Anti-rabbit IgG-(H+L)-HRP conjugate (Biorad Laboratories, CA, USA).

### ELISA

Cell culture supernatants were analyzed for interleukin 10 (IL-10), IL-12/23p40, IL-1α and IL-1RA using ELISA Kit (R&D Systems, Minneapolis, MN, USA) following the manufacturer’s instructions.

### Cytofluorimetric assay

At day 13, BMDCs transfected with 50 nM of miR-369-3p mimic and stimulated with LPS for 24 h were detached from the plate with DPBS (Gibco, NY, USA) supplemented with 0.5 mM EDTA (Thermo Fisher Scientific, MA, USA) washed with DPBS + 0.5% BSA (Sigma Aldrich, St Louis, MO,USA) and stained with CD11c PE-Cy5 (Thermo Fisher Scientific, MA, USA), MHCII APC, CD80 FITC and CD86 FITC (Miltenyi Biotec, Bergisch Gladbach, Germany), according to the manufacture’s instruction. Flow cytometer acquisition was performed using NAVIOS (Beckman Coulter, CA, USA). Flow cytometer analysis was performed using Kaluza Software 1.5.

### Human IBD specimens

Colonic tissue samples from non-inflamed and inflamed regions of intestine were obtained from 11 IBD patients recruited in the Department of Gastroenterology of the National Institute of Gastroenterology “S. de Bellis”. Written informed consent was obtained from all study participants. The study was carried out according to the principles of the Declaration of Helsinki and was approved by the local Institutional Ethics Review Boards (Istituto Tumori Giovanni Paolo II, Bari, Italy). All patients suffering from diabetes, cardiovascular diseases, all cancers, human immunodeficiency virus, hepatitis B and C viruses or all other infectious diseases, pregnancy and all other diseases were excluded from the study. The demographic and clinical characteristic of patients included in the study are summarized in Table 1.

**Table 1:** Demographic and clinical features of IBD patients included in the study.

Variable	Results	
	N (%)	Median (range)
**IBD patients**		
Crohn's disease	7 (63.7)	
Ulcerative colitis	4 (36.3)	
**Male**	9 (81.8)	
**Female**	2 (18.2)	
**Age**		41.9 (30–64)
**Location (n) in CD**		
Ileal	3 (42.8)	
Colonic	2 (28.6)	
Ileal colonic	2 (28.6)	
**Extend in UC**		
Proctis	0	
Left sides colitis	2 (50)	
Extensive colitis	2 (50)	
**Medical treatment**		
Mesalamine	6 (54.5)	
Biologics	5 (45.5)	

Total RNA extraction from tissues was performed with the miRNeasy Mini kit (Qiagen, Hilden, Germany) according to the manufacturer’s instructions. Ten ng of total RNA, including small RNA, were used for the reverse transcription with the TaqMan Advanced miRNA cDNA Synthesis Kit (Thermo Fisher Scientific, MA, USA). Quantitative RT-PCR for miR-369-3p and miR-26a-5p was carried out with TaqMan Advanced miRNA assays and TaqFast Advanced Master mix (Thermo Fisher Scientific, MA, USA). Amplification reactions were performed in triplicate in a final volume of 20 μl on CFX96 System (Biorad Laboratories, CA, USA). For NOS2, IL6 and TNFα gene expression, total RNA was reverse-transcribed with I Script Reverse Transcription Supermix (BioRad Laboratories, CA, USA) according to the manufacturer’s directions. qRT-PCR was performed using the QuantiTect Primer Assay (Qiagen, Hilden, Germany) and SsoAdvanced Universal SYBR Green Supermix (BioRad Laboratories, CA, USA) in 25 µl final volumes. The target signal was normalized to GAPDH gene amplification.

Comparative real-time PCR for miRNAs and mRNAs was performed in triplicate, including no-template controls. Relative expression was calculated using the 2^−ΔCt^ method.

### Bioinformatic and statistical analysis

Putative miRNA gene targets were predicted by miRWalk 2.0 (https://zmf.umm.uni-heidelberg.de/apps/zmf/mirwalk2/)^29^ RNA22 (https://cm.jefferson.edu/rna22/)^30^ algorithms. miRWalk 2.0 algorithm was used to predict miR-369-3p binding sites within CDS region resulting from different algorithms.

Statistical analysis was performed using GraphPad Prism software. Statistical significance of data deriving from different conditions were evaluated with two-way Student’s t test. All values were expressed as the mean ± SEM of data obtained from at least three independent experiments. Result were considered statistically significant at p < 0.05.

## Supplementary information

Supplementary Information
