# Supplementary material for: miR-369-3p modulates inducible nitric oxide synthase and is involved in regulation of chronic inflammatory response
Source: Sci Rep. 2020 Sep 29;10:15942. doi: 10.1038/s41598-020-72991-8 (PMC7525504; doi:10.1038/s41598-020-72991-8)
Supplement: Supplementary file 1 — Supplementary Information [file 41598_2020_72991_MOESM1_ESM.pdf]

SUPPLEMENTARY FIGURE

miR-369-3p modulates inducible nitric oxide synthase and is involved in regulation of chronic inflammatory response

Viviana Scalavino[1*], Marina Liso[1*], Elisabetta Cavalcanti[1], Isabella Gigante[1], Antonio Lippolis[1], Mauro Mastronardi[1], Marcello Chieppa[1] and Grazia Serino[1]

[1]National Institute of Gastroenterology “S. de Bellis”, Research Hospital, 70013, Castellana Grotte (Bari), Italy;

*These authors have equally contributed to this work

Corresponding author: Grazia Serino, National Institute of Gastroenterology “S. de Bellis”, Research Hospital, Via Turi, 27 70013, Castellana Grotte, Bari, Italy.

email: grazia.serino@irccsdebellis.it

ORCID ID: https://orcid.org/0000-0002-2971-0802

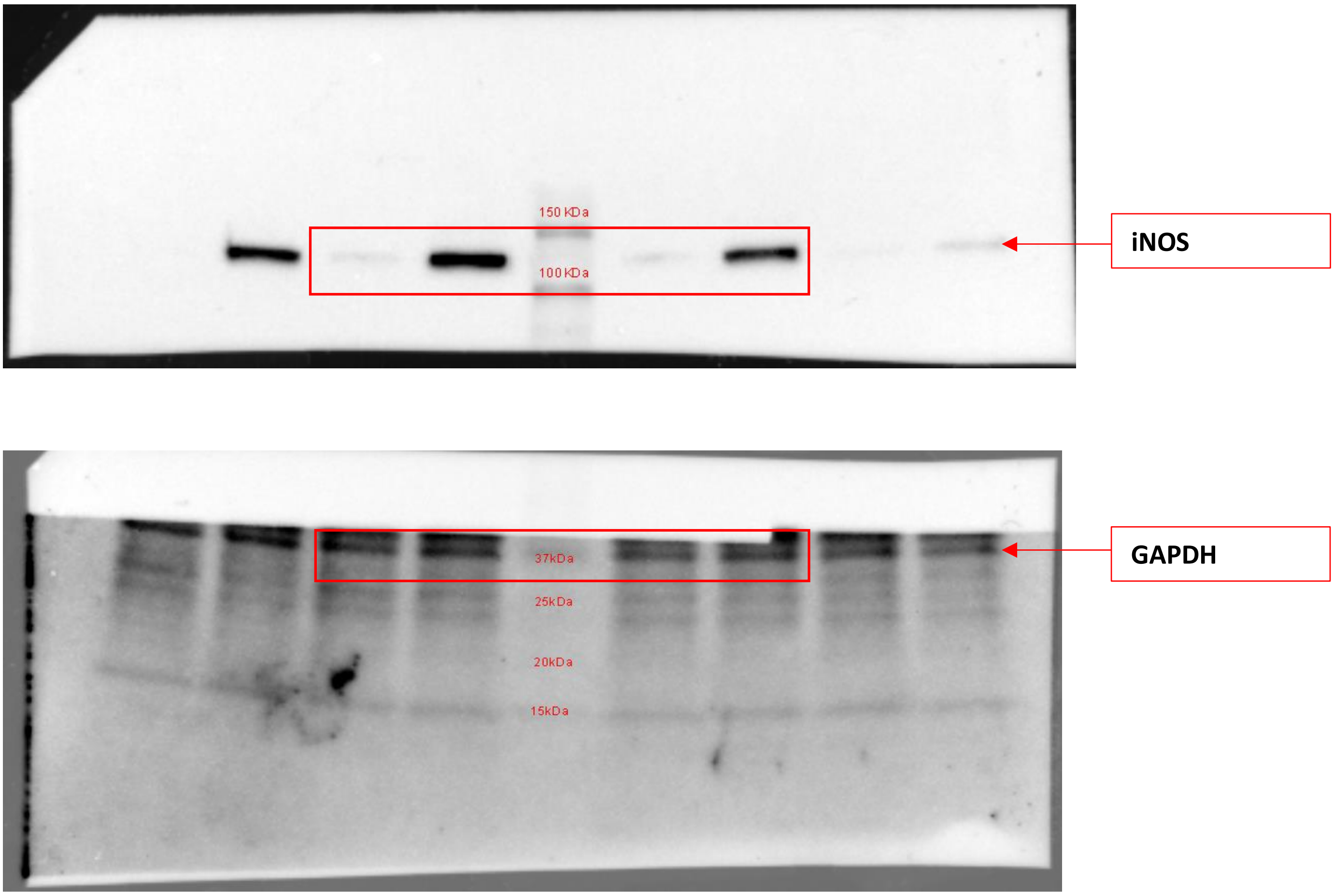

Supplementary Figure 1. Uncropped versions of blots shown in Figure 2B. Blot used for the paper's figure are indicated in red lettering.

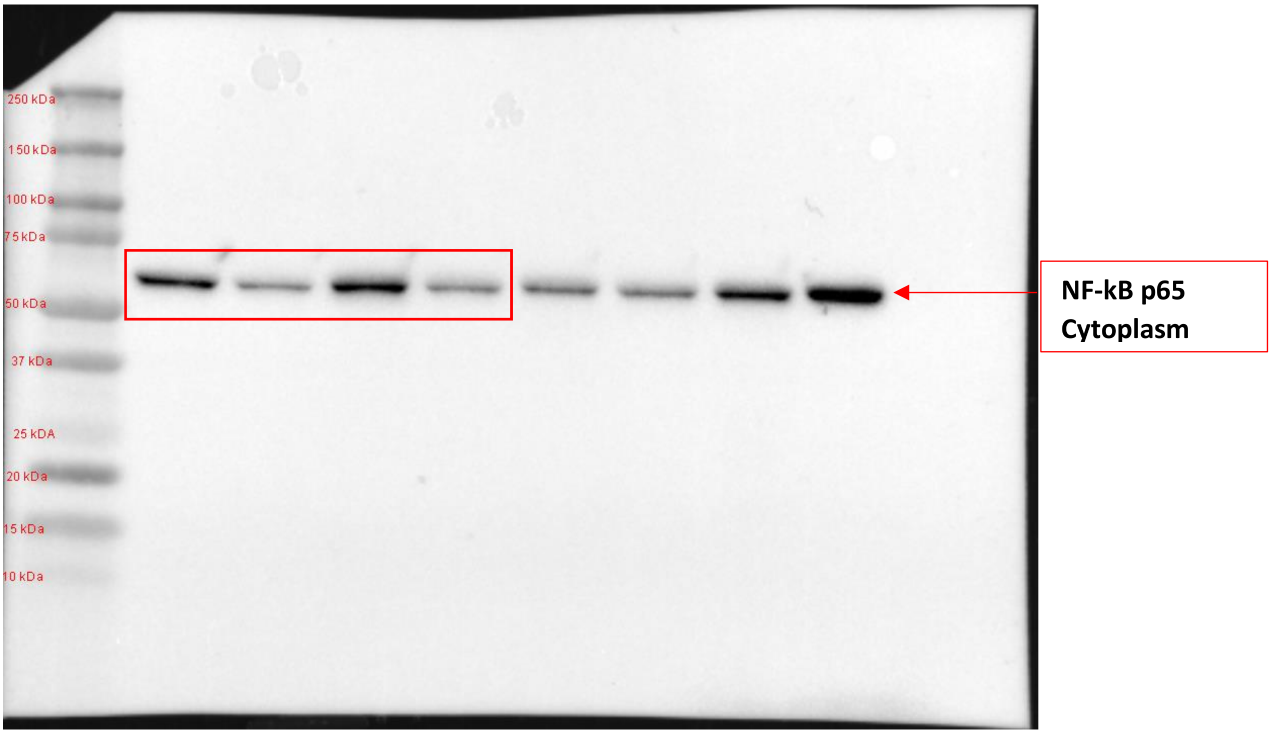
250 kDa
150 kDa
100 kDa
75 kDa
50 kDa
37 kDa
25 kDA
20 kDa
15 kDa
10 kDa
NF-kB p65
Cytoplasm

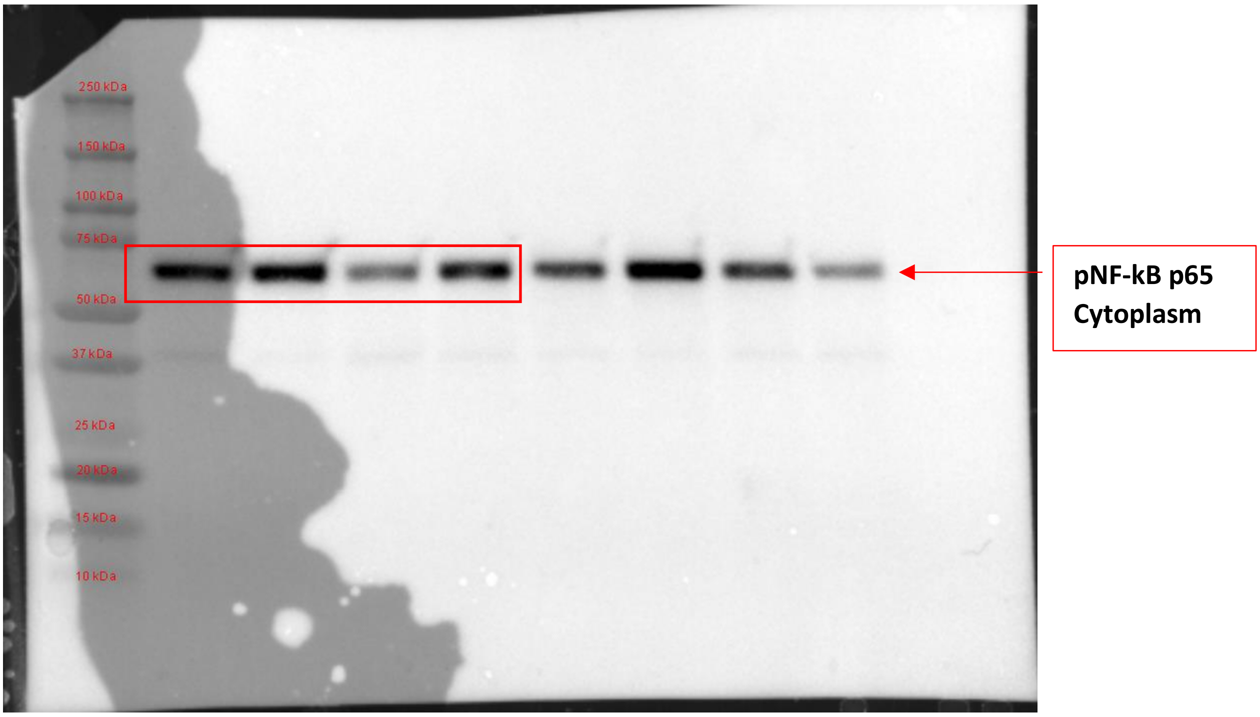
250 kDa
150 kDa
100 kDa
75 kDa
50 kDa
37 kDa
25 kDa
20 kDa
15 kDa
10 kDa
pNF-kB p65
Cytoplasm

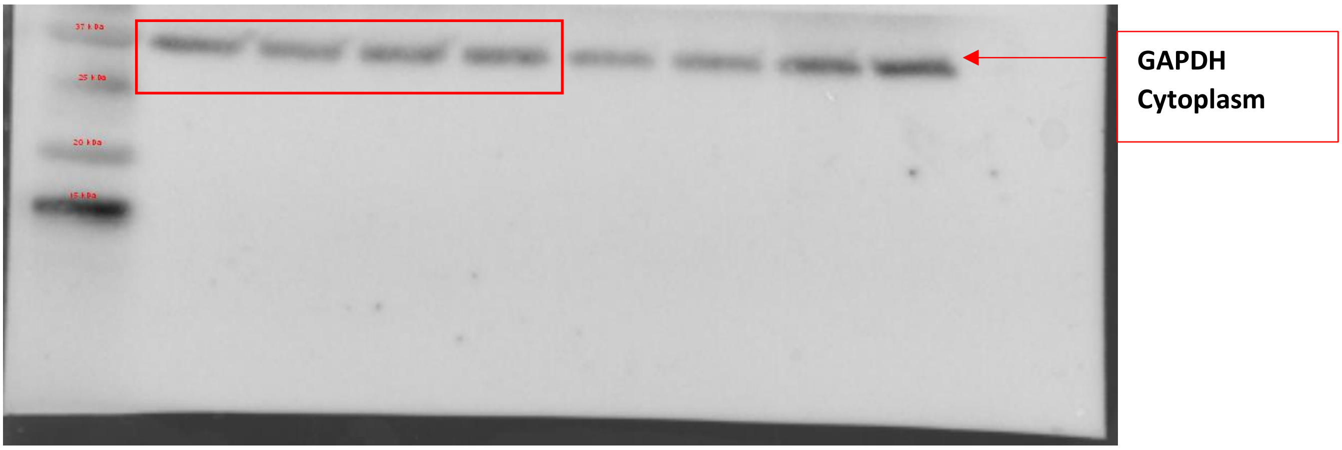
GAPDH
Cytoplasm

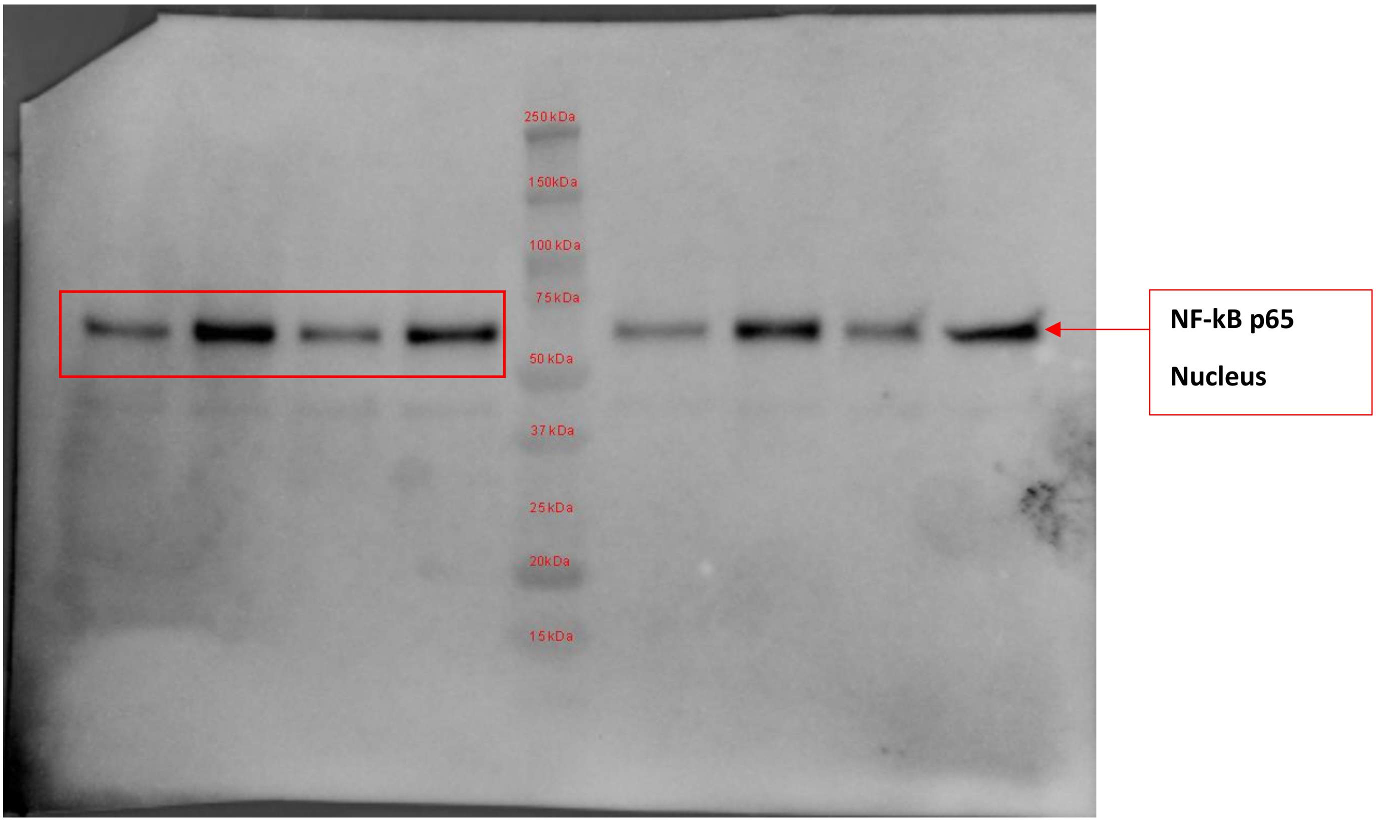
250 kDa
150kDa
100 kDa
75 kDa
50 kDa
37 kDa
25kDa
20kDa
15kDa
NF-kB p65
Nucleus

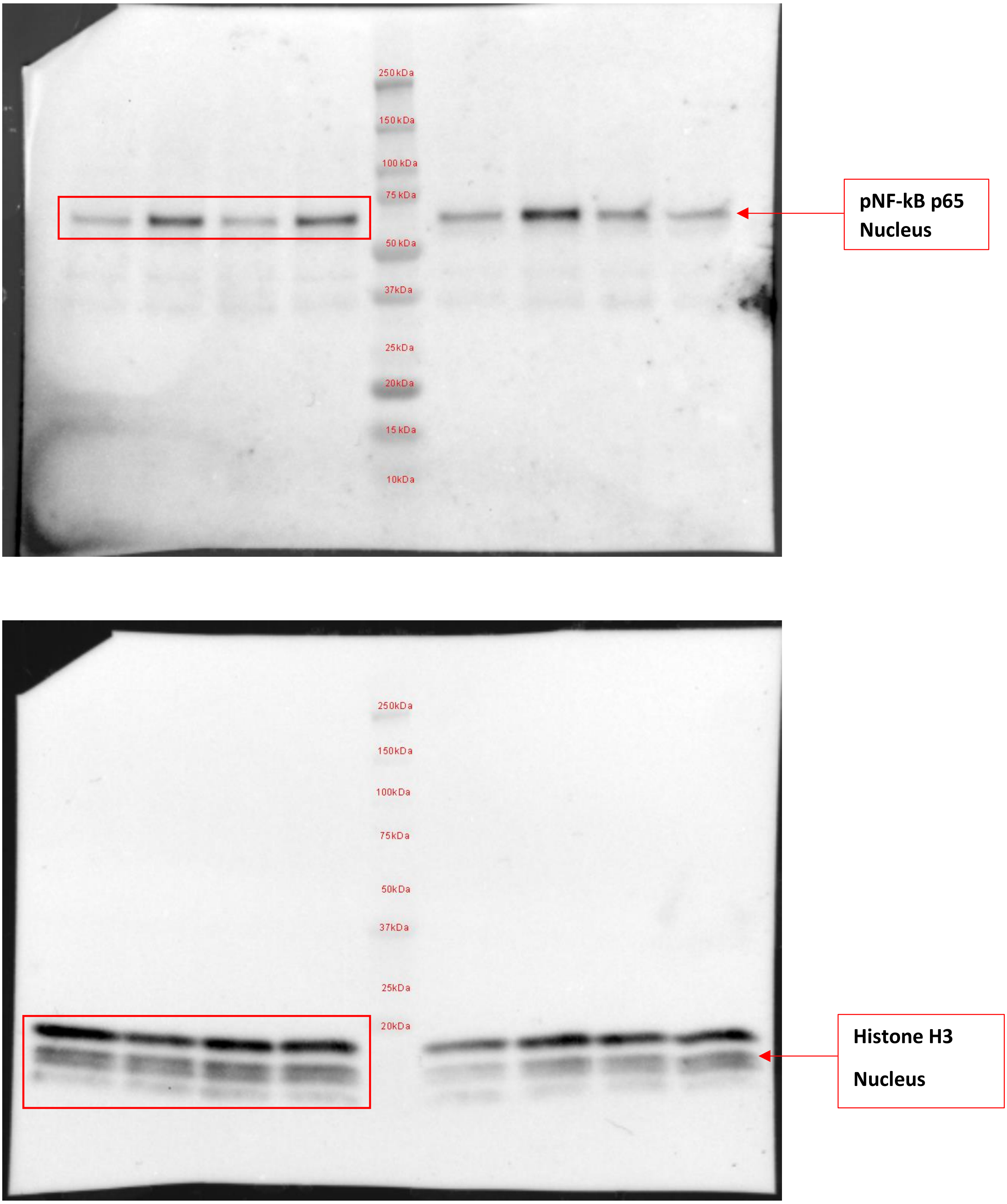

Supplementary Figure 2. Uncropped versions of blots shown in Figure 5A. Blot used for the paper's figure are indicated in red lettering.
